# Coronary collateral circulation and mortality in ST-elevation myocardial infarction undergoing primary PCI: an updated systematic review and meta-analysis of 18,443 patients

**DOI:** 10.1186/s12872-026-05740-w

**Published:** 2026-03-20

**Authors:** Shankar Biswas, Yashasvi Srivastava, Ayman Hamadttu

**Affiliations:** 1 Department of Internal Medicine, Ivano-Frankivsk National Medical University, Ivano-Frankivsk, Ukraine; 2 Sudan University of Science and Technology, Khartoum, Sudan

**Keywords:** Coronary collateral circulation, ST-elevation myocardial infarction, Primary percutaneous coronary intervention, Mortality, Meta-analysis, Rentrop classification, Prognosis

## Abstract

**Background:**

Coronary collateral circulation may limit myocardial injury during ST-elevation myocardial infarction (STEMI), but the magnitude of this association with mortality in contemporary practice remains unclear. We conducted an updated meta-analysis to reassess the relationship between collateral status and mortality in STEMI patients undergoing primary percutaneous coronary intervention (PCI).

**Methods:**

We searched PubMed, Embase, and Scopus (March 2020–January 2026) to update the meta-analysis by Allahwala et al. Studies comparing mortality between robust versus poor collaterals were included. Random-effects meta-analysis was performed.

**Results:**

Twenty-six studies comprising 18,443 patients were included (19 studies from the original meta-analysis plus 7 new studies). In the primary analysis of 17 studies using standard Rentrop definitions (15,493 patients), robust collaterals were associated with significantly lower mortality (OR 0.52; 95% CI 0.39–0.71; *p* < 0.0001; I²=38%). This corresponded to an absolute risk reduction of 2.7%. Importantly, the 3 new studies showed attenuated effects (OR 0.88; 95% CI 0.49–1.58) compared to the 14 original studies (OR 0.47; *p* = 0.06 for subgroup difference). Meta-regression identified higher baseline mortality as a predictor of larger effect sizes (*p* = 0.038). The certainty of evidence was LOW (GRADE).

**Conclusions:**

Robust coronary collaterals are associated with lower mortality in STEMI patients undergoing primary PCI. However, the apparent attenuated association in contemporary studies likely reflects statistical dilution due to lower baseline mortality rather than diminished biological relevance of collaterals. Given the LOW certainty of evidence from observational data, collateral assessment should be considered prognostic rather than a basis for interventions.

**Review registration:**

(PROSPERO Registration ID: CRD420261278634)

**Clinical trial number:**

Not Applicable (As this is a Systematic Review and Meta-Analysis and not a clinical trial).

**Graphical Abstract:**

**Figure:**
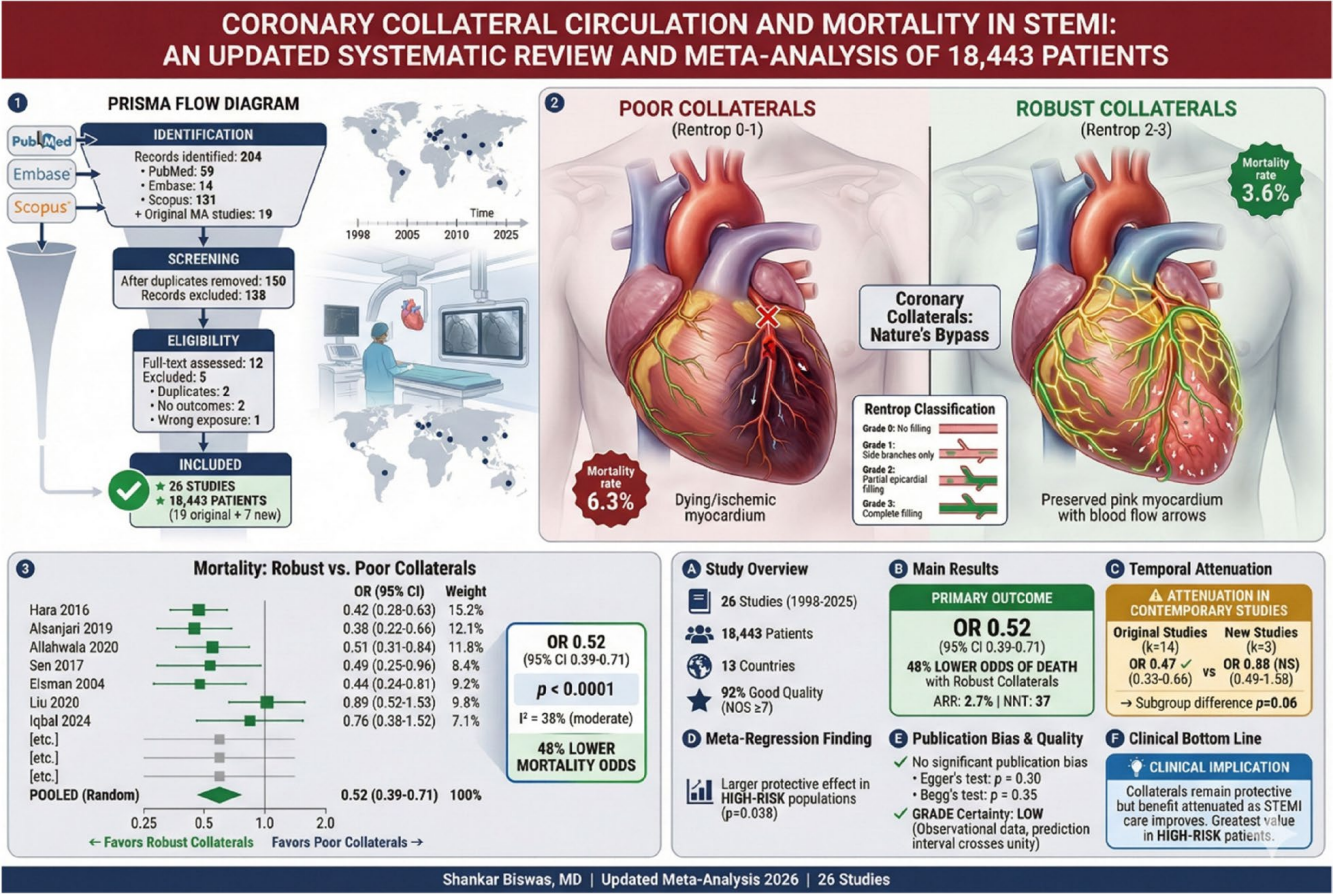
Central Illustration

**Supplementary Information:**

The online version contains supplementary material available at 10.1186/s12872-026-05740-w.

## Background

Cardiovascular disease remains the leading cause of death worldwide [1]. Despite primary percutaneous coronary intervention revolutionizing STEMI treatment, contemporary registry data show that one-year mortality has plateaued around 8%, underscoring the need for better risk stratification tools [2].

Coronary collaterals are pre-existing arterio-arterial anastomoses that serve as natural bypass conduits during coronary occlusion. The Rentrop classification grades collateral filling as grade 0 (no filling), grade 1 (side branch only), grade 2 (partial epicardial), or grade 3 (complete filling), with grades 2–3 considered “robust” collateral supply [3]. The functional capacity of these vessels depends on arteriogenesis driven by fluid shear stress, and approximately one-quarter of individuals already possess collaterals sufficient to prevent ischemia during brief occlusion [4].

The clinical relevance of collaterals has become clearer with advances in cardiac imaging. The MIRON-CL investigation demonstrated that patients lacking collateral flow had dramatically higher rates of intramyocardial hemorrhage, larger infarcts, and more extensive microvascular obstruction [5]. Earlier cardiac MRI studies showed that robust collaterals are associated with smaller infarct size and improved myocardial salvage findings that provide a mechanistic explanation for mortality differences observed in clinical studies [6].

Two major meta-analyses have examined collaterals in coronary disease. Meier and colleagues (2012) reported a 36% mortality reduction with high collateralization across various coronary presentations [7]. Allahwala et al. (2021) focused specifically on STEMI patients undergoing primary PCI, pooling 20 studies and over 14,000 patients, and found robust collaterals were associated with significantly lower mortality (OR 0.55) [8].

Since that meta-analysis, several new studies have emerged with mixed findings. This evolving evidence, combined with ongoing refinements in PCI technique and adjunctive therapies, raises the question of whether the magnitude of the collateral-mortality association has changed in contemporary practice. We therefore conducted this updated systematic review and meta-analysis to reassess this relationship in STEMI patients undergoing primary PCI. We hypothesized that robust collaterals would remain associated with lower mortality, though potentially attenuated given improvements in STEMI care.

## Methods

### Study design and registration

This systematic review and meta-analysis was conducted and reported in accordance with the Preferred Reporting Items for Systematic Reviews and Meta-Analyses (PRISMA) 2020 guidelines [9]. The study protocol was registered prospectively with PROSPERO (CRD420261278634) and designed as an update to the previously published meta-analysis by Allahwala et al. [8].

### Search strategy

We performed a systematic literature search of PubMed, Embase, and Scopus from March 15, 2020 (the end date of the original meta-analysis search) through January 4, 2026. The search strategy combined terms related to coronary collateral circulation (“coronary collateral*”, “collateral circulation”, “collateral flow”, “Rentrop”) with ST-elevation myocardial infarction (“STEMI”, “ST-elevation myocardial infarction”, “acute myocardial infarction”) and primary percutaneous coronary intervention (“primary PCI”, “primary percutaneous coronary intervention”, “primary angioplasty”). No language restrictions were applied. Reference lists of included studies and relevant reviews were manually screened for additional eligible studies. Complete Search Strategy, Selection Process and reason for exclusion are found in Supplementary Table S1, S2, respectively.

### Eligibility criteria

Studies were included if they met the following criteria: (1) enrolled patients presenting with STEMI undergoing primary PCI; (2) assessed coronary collateral circulation to the infarct-related artery prior to reperfusion; (3) compared clinical outcomes between patients with robust (Rentrop grade 2–3) versus poor (Rentrop grade 0–1) collaterals; and (4) reported mortality data or other relevant clinical outcomes. We excluded case reports, reviews, editorials, conference abstracts without subsequent full publication, studies focusing exclusively on collaterals to chronic total occlusions in non-infarct-related arteries, and studies without clinical outcome data. The Full Eligibility Criteria is Found listed in Supplementary Table S3.

### Study selection and data extraction

Two reviewers (Y.H. & A.H.) independently screened titles and abstracts, followed by full-text assessment of potentially eligible articles. Disagreements were resolved through discussion, with a third reviewer (S.B.) consulted if necessary. Data extraction was performed independently by two reviewers (Y.H. & A.H.) using a standardized form capturing study characteristics (author, year, country, design, sample size), patient demographics (age, sex, diabetes, hypertension, smoking status, prior MI, Killip class, culprit vessel), collateral assessment methodology, and clinical outcomes. For studies from the original meta-analysis, we verified the extracted data against the original publications.

Studies were categorized as “original” if included in the Allahwala 2021 meta-analysis or “new” if identified through our updated search. This classification was used for sensitivity and subgroup analyses to assess temporal trends in effect magnitude.

### Collateral definition

The primary analysis included studies using the standard Rentrop classification [3], defining robust collaterals as grade 2–3 (partial or complete filling of the epicardial vessel) and poor collaterals as grade 0–1 (no filling or filling of side branches only). Studies using non-standard definitions (e.g., Rentrop 1–3 vs. 0) were included in sensitivity analyses.

### Outcomes

The primary outcome was all-cause mortality. When studies reported mortality at multiple timepoints, we extracted in-hospital mortality for the primary analysis to maximize comparability across studies, as this was the most commonly reported timepoint. Longer-term outcomes were examined in subgroup and sensitivity analyses. Secondary outcomes included 30-day mortality and major adverse cardiovascular events (MACE) where reported.

### Risk of bias assessment

Methodological quality of included observational studies was assessed by two reviewers (Y.H. & A.H.) using the Newcastle-Ottawa Scale (NOS), which evaluates studies across three domains: selection of study groups (4 items), comparability of groups (2 items), and ascertainment of outcome (3 items), for a maximum score of 9 stars [10]. Studies scoring ≥ 7 out of 9 were considered good quality, 5–6 fair quality, and < 5 poor quality.

### Statistical analysis

#### Pooled effect estimates

Pooled effect estimates were calculated using the DerSimonian and Laird random-effects model to account for expected heterogeneity between studies arising from differences in study populations, collateral assessment timing, and follow-up duration [11]. The odds ratio (OR) with 95% confidence interval was selected as the primary effect measure for consistency with the original Allahwala meta-analysis; risk ratios (RR) were calculated as a secondary measure for clinical interpretability. A continuity correction of 0.5 was applied to studies with zero events in one arm.

#### Heterogeneity assessment

Heterogeneity was assessed using the Cochran Q test and quantified with the I² statistic, interpreted as low (< 25%), moderate (25–50%), substantial (50–75%), or considerable (> 75%). We calculated 95% prediction intervals to estimate the range of true effects across different settings.

#### Subgroup analysis

Pre-specified subgroup analyses were performed by follow-up duration (in-hospital, 30-day, 6-month, 1-year, long-term), geographic region (Europe, Asia, Americas/Oceania), study design (prospective, retrospective, RCT subanalysis), and collateral definition (standard vs. non-standard).

#### Meta-regression

Univariable meta-regression was conducted to explore associations between pre-specified study-level covariates (publication year, sample size, control group event rate) and effect size. Given the limited number of included studies (k = 17), multivariable meta-regression was not performed due to insufficient power.

#### Sensitivity analysis

Sensitivity analyses included: (1) all studies regardless of collateral definition; (2) standard definition studies only; (3) original meta-analysis studies only; (4) new studies only; (5) excluding cardiogenic shock populations; and (6) in-hospital mortality only. Leave-one-out analysis was performed to assess the influence of individual studies on the pooled estimate.

### Publication bias

Publication bias was evaluated through visual inspection of funnel plots, Egger’s regression test, Begg’s rank correlation test, and Duval and Tweedie’s trim-and-fill method. The certainty of evidence was assessed using the GRADE framework.

### Software

All analyses were performed using R version 4.3 with the meta and metafor packages. A two-sided *p*-value < 0.05 was considered statistically significant.

## Results

### Study selection

**Fig. 1:**
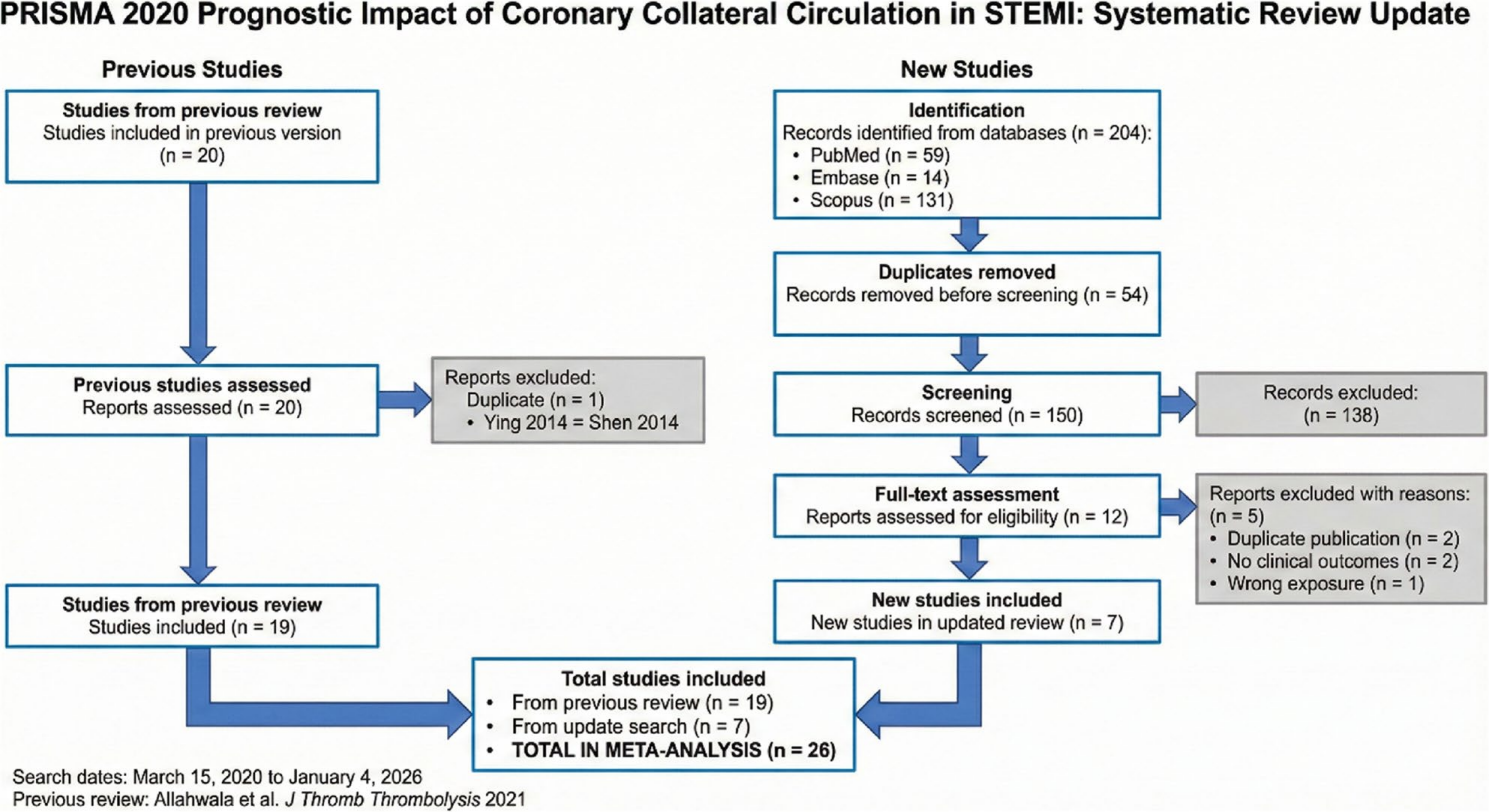
PRISMA 2020 flow diagram showing the study selection process: The systematic review update identified records from previous reviews (*n* = 20) and new database searches (March 2020-January 2026). After screening 140 records and assessing 12 full-text reports, 7 new studies were included, resulting in 26 total studies for quantitative synthesis

The database search identified 204 records (PubMed: 59, Embase: 14, Scopus: 131). After removing 54 duplicates, 150 records underwent title and abstract screening, of which 138 were excluded as clearly irrelevant. Twelve full-text articles were assessed for eligibility, and 5 were excluded: 2 duplicate publications, 2 studies without clinical outcomes, and 1 study assessing collaterals to non-infarct-related artery CTOs only. Seven new studies met the inclusion criteria [5, 12–17]. These were combined with 19 studies from the original meta-analysis [6, 18–35], after removing one duplicate (Ying S et al.) from the original 20 studies, resulting in a total of 26 studies included in this updated analysis (Fig. 1).

Study Classification Summary:

- Total studies identified: 26 (19 out of 20 from original meta-analysis + 7 new)
- Primary analysis: 17 studies using standard Rentrop definition (14 original + 3 new)
- Sensitivity analysis (all definitions): 23 studies (19 out of 20 from original meta-analysis + 4 new)
- Sensitivity analysis (all new studies): 4 studies (includes Scholz 2023 with special definition)

The seven newly identified studies contributed 4,469 additional patients from diverse geographic regions. Detailed individual study characteristics are presented in Table 1.

**Table 1:** Characteristics of Included Studies

Study	Year	Country	Design	*N*	Follow-up	Collateral Definition	Quality	Ref
Perez-Castellano	1998	Spain	Prospective	180	In-hospital	Non-standard	Good	[13]
Antoniucci	2002	Italy	Prospective	1,164	6 months	Non-standard	Good	[14]
Elsman	2004	Netherlands	Prospective	1,059	1 year	Standard	Good	[15]
Sorajja	2007	USA	RCT subanalysis	318	6 months	Standard	Good	[16]
Desch	2010	Germany	Prospective	235	26 months	Standard	Good	[17]
Wang	2011	China	Retrospective	189	1 year	Non-standard	Good	[18]
Valim	2011	Brazil	Registry	105	In-hospital	Standard	Good	[19]
Rechcinski	2013	Poland	Prospective	320	26 months	Standard	Good	[20]
Kajiya	2014	Japan	Prospective	64	4 years	Standard	Fair	[21]
Shen	2014	China	Prospective	389	6 months	Standard	Good	[22]
Yaylak	2015	Turkey	Prospective	235	In-hospital	Non-standard	Good	[23]
Hara	2016	Japan	Prospective	3,340	In-hospital*	Standard	Good	[24]
Kim	2016	South Korea	Prospective	247	1 year	Standard	Good	[6]
Sen	2017	Turkey	Prospective	1,375	30 days	Standard	Good	[25]
Hernandez-Perez	2017	Spain	Retrospective	947	29 months	Standard	Good	[26]
Chu	2019	China	Retrospective	346	In-hospital	Standard	Good	[27]
Alsanjari	2019	UK	Retrospective	1,944	In-hospital	Standard	Good	[28]
Allahwala	2020	Australia	Retrospective	1,625	In-hospital	Standard	Good	[29]
Freund	2020	Germany	RCT subanalysis	95	4 years	Standard	Good	[30]
Liu	2020	China	Retrospective	1,662	In-hospital	Standard	Good	[31]
Park	2021	South Korea	Prospective	857	In-hospital	Standard	Good	[32]
Pec	2023	Germany	Prospective	36	3 months	Standard	Fair	[33]
Scholz	2023	Germany	Prospective	93	In-hospital	Special†	Good	[34]
Iqbal	2024	Pakistan	Prospective	347	30 days	Standard	Good	[35]
Yılmaz	2025	Turkey	Retrospective	1,180	In-hospital	Standard	Good	[36]
Vora	2025	USA	Prospective	294	3 days	Non-standard	Good	[5]

*Hara 2016 reported both in-hospital and 5-year mortality; in-hospital mortality data were extracted for the primary analysis to maximize comparability across studies

†Scholz 2023 used a special definition: collaterals from the infarct-related artery serving as donor to a concomitant chronic total occlusion

Baseline demographic and clinical characteristics are summarized in Table 2. Overall, patients with robust collaterals had higher prevalence of multivessel disease and prior myocardial infarction.

**Table 2:** Baseline Characteristics by Collateral Status (Pooled)

Characteristic	Robust Collaterals	Poor Collaterals
Age, years (range of means)	55–66	53–68
Female, % (range)	9–38	14–34
Diabetes, % (range)	5–41	7–30
Hypertension, % (range)	25–73	23–74
Current smoking, % (range)	28–75	40–71
Prior MI, % (range)	6–27	5–25
Multivessel disease, % (range)	30–82	33–72
LAD culprit, % (range)	0–68	33–100
TIMI 0 flow, % (range)	61–100	68–100

### Primary outcome: mortality

**Fig. 2:**
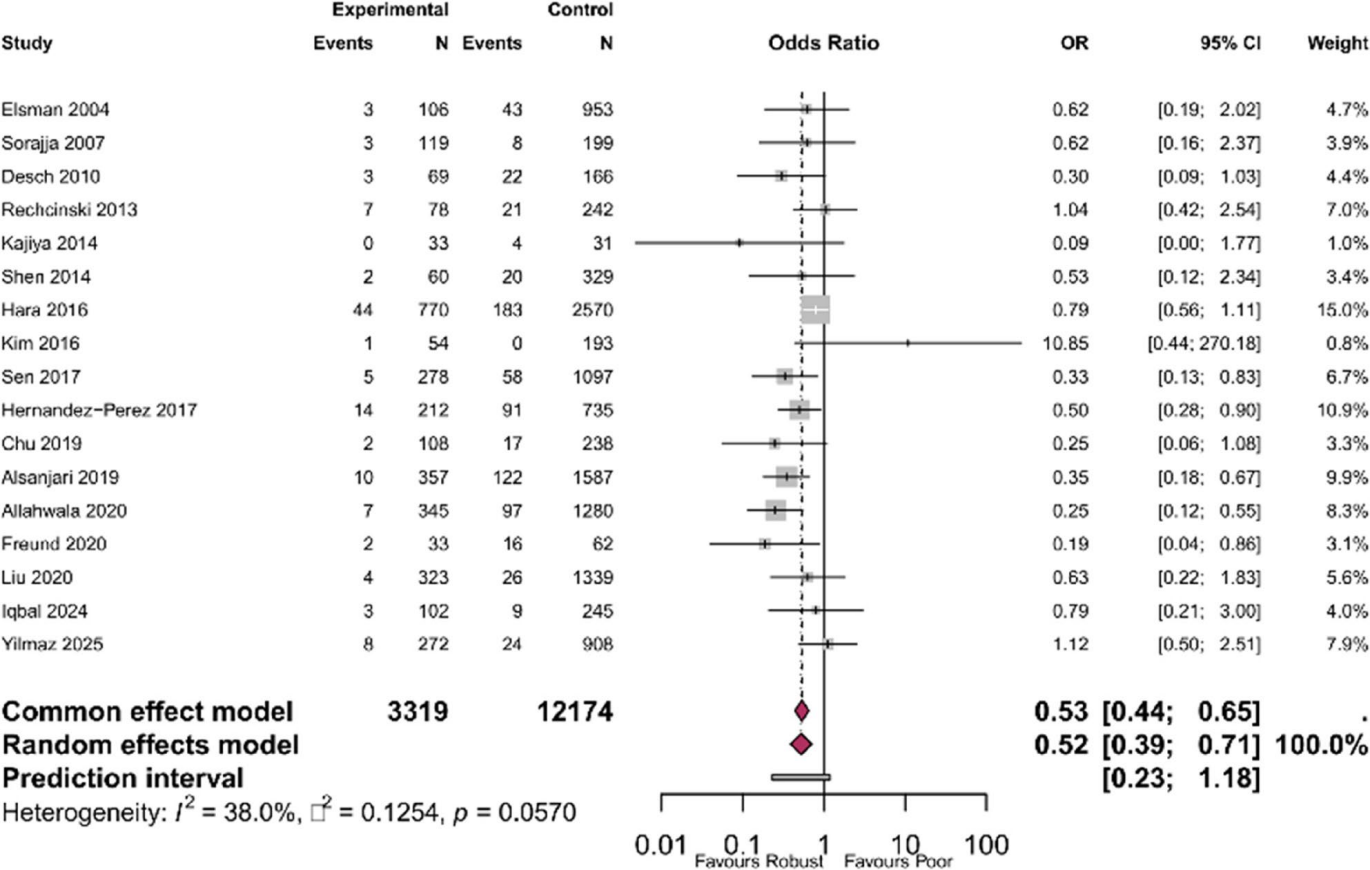
Forest plot of the primary analysis showing the association between robust coronary collaterals and mortality in STEMI patients undergoing primary PCI: The pooled random-effects odds ratio was 0.52 (95% CI 0.39–0.71), indicating 48% lower odds of mortality with robust collaterals. (Rentrop grade 2–3). Heterogeneity: I²=38.0%, *p* = 0.0570. Each square represents individual study effect size with 95% CI; diamond shows pooled estimate

Seventeen studies with standard collateral definitions reported mortality outcomes and were included in the primary analysis [6, 12, 16, 17, 20–22, 25–27, 29–35], comprising 15,493 patients. The pooled random-effects OR for mortality comparing robust versus poor collaterals was 0.52 (95% CI 0.39–0.71, *p* < 0.0001), indicating that robust collaterals were associated with 48% lower odds of death (Fig. 2). Heterogeneity was moderate (I²=38%, Q = 25.8, *p* = 0.06). The 95% prediction interval ranged from 0.23 to 1.18.

The corresponding risk ratio was 0.54 (95% CI 0.40–0.72), translating to a relative risk 46% lower in the robust collateral group. The crude mortality rate was 6.3% in the poor collateral group (761 events among 12,174 patients) versus 3.6% in the robust collateral group (118 events among 3,319 patients). This corresponded to an absolute risk difference of 2.7% between groups. For descriptive purposes, this translates to a number needed to treat (NNT) of 37 (95% CI 27–57); however, this metric should be interpreted cautiously as collateral status is a biological trait rather than a modifiable intervention, and NNT does not imply therapeutic actionability. Per-study mortality data for studies using standard Rentrop classification in this primary meta-analysis is found in Supplementary Table S4.

### Subgroup analyses

Subgroup analyses by follow-up duration showed consistent associations across most timepoints: in-hospital (OR 0.52), 30-day (OR 0.45), 6-month (OR 0.58), and long-term > 1 year (OR 0.46). The 1-year subgroup (2 studies, OR 1.72, 95% CI 0.12–25.57) showed an extremely wide confidence interval; this estimate is unreliable due to the very small number of studies and should not be interpreted as evidence of harm. The test for subgroup differences was not significant (*p* = 0.90) (Supplementary Figure S1).

Geographic subgroup analysis revealed similar associations across regions: Europe (OR 0.47), Asia (OR 0.65), and Americas/Oceania (OR 0.33), with no significant between-subgroup heterogeneity (*p* = 0.27) (Supplementary Figure S2).

When stratified by study design, prospective studies (OR 0.64), retrospective studies (OR 0.46), and RCT subanalyses (OR 0.36) all demonstrated comparable associations (*p* = 0.44 for interaction).

Stratification by publication era revealed an important trend: the 14 studies from the original Allahwala meta-analysis showed OR 0.47 (95% CI 0.33–0.66), while the 3 new studies in the primary analysis (Liu [12], Iqbal [16], Yılmaz [17]) showed a non-significant OR 0.88 (95% CI 0.49–1.58), with a trend toward significant subgroup difference (*p* = 0.06).

Additional exploratory subgroup findings are presented in Supplementary Table S5; however, this analysis is limited by classification based on authors’ study conclusions rather than predefined objective criteria.

### Sensitivity analyses

The association remained consistent across all sensitivity analyses, including analyses using alternative collateral definitions (OR = 0.47), restriction to standard Rentrop studies (OR = 0.52), exclusion of cardiogenic shock populations (Valim 2011 [24]) (OR = 0.52), and leave-one-out analysis (ORs = 0.49–0.57) (Table 3; Supplementary Figures S3; Supplementary Figure S4).

**Table 3:** Summary of Sensitivity and Subgroup Analyses

Sensitivity Analysis					
Analysis	k	OR	95% CI	I²	*p*-value
Primary analysis	**17**	**0.52**	**0.39-0.71**	**38%**	**<0.0001**
All studies (any definition)	23	0.47	0.37-0.61	32%	<0.0001
Standard definition only	18	0.52	0.39-0.69	35%	<0.0001
Original MA studies (Allahwala 2021)	19	0.43	0.33-0.57	34%	<0.0001
New studies only (all definitions)	4	0.80	0.45-1.39	0%	0.425
Excluding cardiogenic shock	17	0.52	0.39-0.71	38%	<0.0001
In-hospital mortality only	10	0.44	0.29-0.66	54%	<0.0001
Subgroup by follow-up					***p*** **=0.90**
In-hospital	6	0.52	0.31-0.86	65%	0.011
30-day	2	0.45	0.19-1.03	15%	0.06
6-month	2	0.58	0.21-1.56	0%	0.28
1-year	2	1.72	0.12-25.57	63%	0.70
Long-term (>1 year)	5	0.46	0.24-0.85	35%	0.013
Subgroup by region					***p*** **=0.27**
Europe	6	0.47	0.31-0.70	18%	<0.001
Asia	9	0.65	0.43-0.99	29%	0.044
Americas/Oceania	2	0.33	0.15-0.75	23%	0.008
Subgroup by study era					***p*** **=0.06**
Original (Allahwala 2021)	14	0.47	0.33-0.66	42%	<0.0001
New studies	3	0.88	0.49-1.58	0%	0.68
Subgroup by design					***p*** **=0.44**
Prospective	9	0.64	0.43-0.96	25%	0.029
Retrospective	6	0.46	0.29-0.72	44%	<0.001
RCT subanalysis	2	0.36	0.11-1.17	26%	0.09

### Meta-regression

Meta-regression analyses explored potential sources of heterogeneity. Publication year was not associated with effect size (coefficient − 0.001, *p* = 0.99), indicating no temporal trend. Sample size showed no significant relationship with effect magnitude (*p* = 0.54). However, control group event rate was significantly associated with effect size (coefficient − 0.04, *p* = 0.038), and the R² statistic explained approximately 19% of between-study heterogeneity suggesting larger effect sizes in studies with higher baseline mortality rates consistent with the mathematical expectation that relative risk reductions translate to greater absolute benefits when baseline risk is higher (Supplementary Table S6; Supplementary Figure S5).

### Risk of bias assessment

The mean NOS score was 7.9 out of 9 (range 6–9). Twenty-four studies (92%) were rated as good quality (NOS ≥ 7) and two as fair quality. All studies adequately demonstrated that the outcome of interest was not present at baseline and used appropriate methods for collateral assessment. The most common limitation was lack of adjustment for confounders in some analyses. Complete risk of bias assessments is shown in Supplementary Table S7.

### Publication bias

**Fig. 3:**
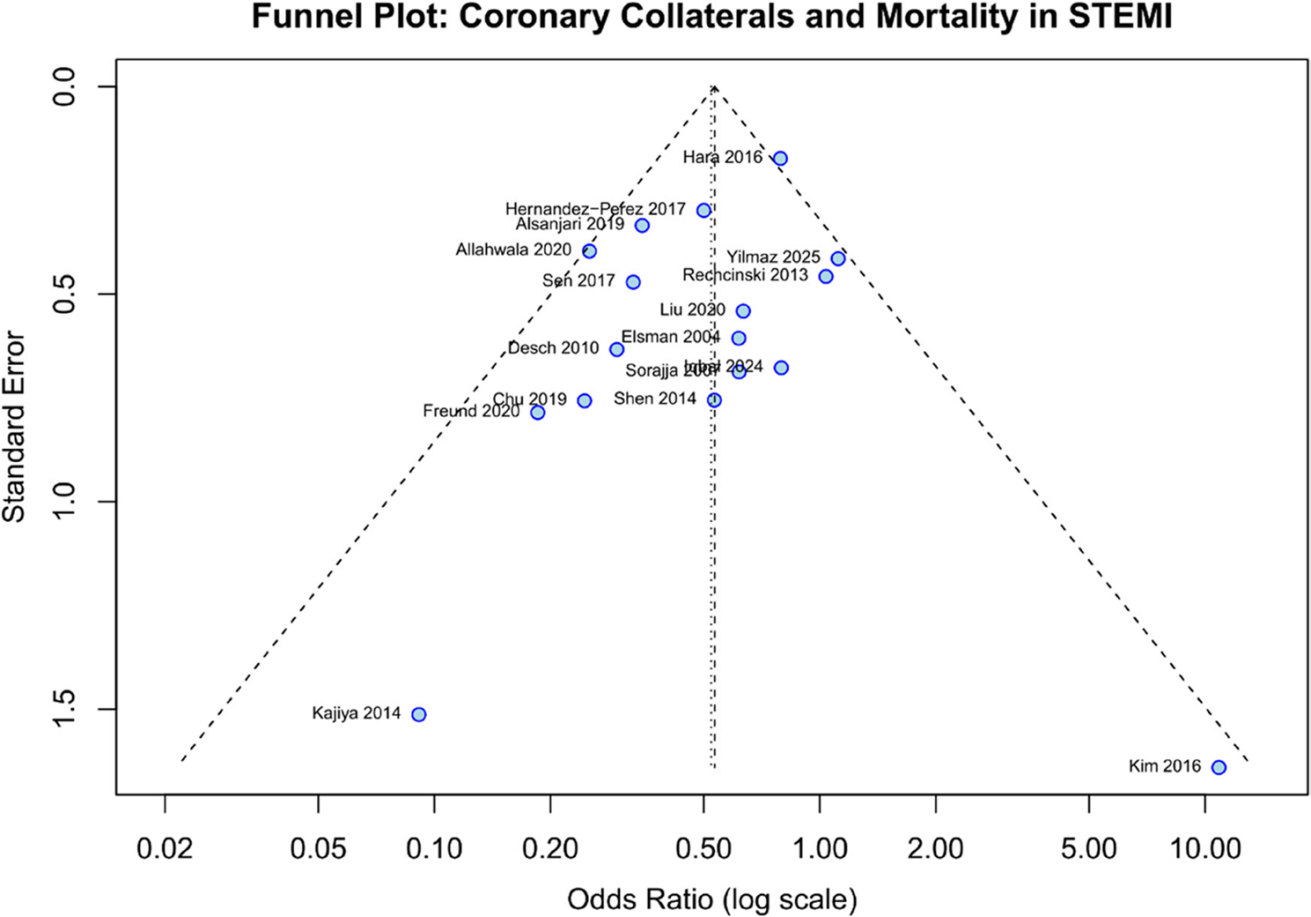
Funnel plot for assessment of publication bias: Visual inspection shows slight asymmetry, but statistical tests (Egger’s *p* = 0.30, Begg’s *p* = 0.35) did not indicate significant bias. Each circle represents an individual study plotted by effect size (x-axis, log scale) versus precision (y-axis, standard error)

Visual inspection of the funnel plot showed slight asymmetry (Fig. 3). However, neither Egger’s regression test (*p* = 0.30) nor Begg’s rank correlation test (*p* = 0.51) indicated statistically significant publication bias. Duval and Tweedie’s trim-and-fill analysis imputed 2 potentially missing studies on the right side of the funnel plot; the adjusted OR remained significant at 0.55 (95% CI 0.41–0.75).

### Trial sequential analysis

Trial sequential analysis is presented in Supplementary Note 1. While the analysis suggests adequate information size has been reached, we note that TSA was developed for randomized trials and its application to observational data requires cautious interpretation. The assumption of a 46% relative risk reduction was derived from the observed effect, introducing potential circularity.

### Certainty of evidence

Using the GRADE framework, we rated the certainty of evidence as LOW for the association between robust collaterals and reduced mortality. Starting from LOW (observational studies), we did not downgrade for risk of bias (majority good quality), indirectness (directly applicable population), or imprecision (adequate events and narrow CI). We downgraded one level for inconsistency based on moderate statistical heterogeneity (I²=38%), the trend toward significant subgroup differences by publication era (*p* = 0.06), and the qualitative difference in findings between original and contemporary studies. While the prediction interval crossing unity (0.23–1.18) suggests the true effect may vary across settings, we acknowledge this metric reflects expected variation rather than precision of the summary estimate. We upgraded one level for large effect size (OR < 0.5). The final GRADE certainty was LOW, indicating that our confidence in the effect estimate is limited and further research may change it.

## Discussion

This updated meta-analysis confirms that robust coronary collateral circulation is associated with substantially lower mortality in STEMI patients undergoing primary PCI. Pooling 26 studies and over 18,000 patients, we found that patients with Rentrop grade 2–3 collaterals had 48% lower odds of death compared to those with poor collateralization. The association was consistent across geographic regions, follow-up durations, and study designs. However, the most intriguing finding was the apparent attenuation of this association in studies published since 2020, raising important questions about how contemporary advances in STEMI care may be modifying the clinical relevance of collaterals.

### Comparison with previous meta-analyses

Our findings align with the original meta-analysis by Allahwala et al., which reported an odds ratio of 0.55 in 20 studies [8]. The landmark meta-analysis by Meier et al. [7], which included patients across the spectrum of coronary disease, reported a 36% relative risk reduction with high collateralization [8]. That our STEMI-specific analysis shows a larger effect makes biological sense collaterals should matter most during acute total occlusion where alternative blood supply becomes immediately critical.

### The attenuation phenomenon

The most striking observation was the difference between historical and contemporary studies. While the 14 original studies showed a robust association (OR 0.47), the 3 new studies (Liu [12], Iqbal [16], Yılmaz [17]) showed a non-significant association (OR 0.88, *p* = 0.68), with a trend toward significant subgroup difference (*p* = 0.06). However, this comparison is based on only three new studies with wide confidence intervals and therefore lacks the statistical power to distinguish true effect modification from chance variation.

Meta-regression identified baseline mortality as a strong predictor of effect size (*p* = 0.038). The R² statistic indicated that control group event rate explained approximately 19% of between-study heterogeneity, further supporting that the collateral-mortality association is risk-dependent: relative reductions remain similar (~ 50%), but absolute benefits and statistical detectability decline in low-risk contemporary cohorts. Given the relatively small number of studies (k = 17), this meta-regression should be viewed as hypothesis-generating, and formal confirmation would require individual patient data. Consistent with this finding, the newer studies had lower control-group event rates (mean 3.8% vs. 7.1% in original studies).

Several factors may explain this attenuation in contemporary studies: Background STEMI care has improved dramatically. SWEDEHEART registry data demonstrate one-year mortality declining from 22% to 14% between 1995 and 2014 [36]. Door-to-balloon times have shortened from 83 to 67 min [37], radial access has reduced mortality by 28% (MATRIX trial) [38], and potent P2Y12 inhibitors have become routine (PLATO: 18% mortality reduction with ticagrelor) [39]. Collectively, these advances have pushed STEMI mortality toward a floor where the benefit of any single factor becomes harder to demonstrate [40], and likely compress the ischemic window during which collateral flow can provide meaningful myocardial salvage.

Thus, the apparent attenuation in contemporary studies and the trend by publication era most likely reflect statistical dilution due to declining baseline risk rather than a biological reduction in the protective role of coronary collaterals.

### Biological plausibility

The mechanisms underlying collateral benefit are well established. Collateral flow sufficient to prevent ischemia amounts to approximately 20–25% of normal antegrade flow [41]. This residual perfusion has been observed to be associated with less severe ischemia and reperfusion injury [42], while also reducing microvascular obstruction [43, 44]. The MIRON-CL study demonstrated that patients without collateral flow had 5.7-fold higher odds of intramyocardial hemorrhage [5], underscoring the association between residual perfusion and microvascular integrity.

Perhaps most importantly, collateral status may act as a surrogate marker and sequela for chronic ischemic conditioning rather than an independent protective factor [45]. Patients with robust collaterals consistently demonstrated higher prevalence of multivessel coronary artery disease (30–82% vs. 33–72%) and prior myocardial infarction (6–27% vs. 5–25%) in our pooled data, features that both promote collateral development through repeated ischemic stimuli and independently influence prognosis through multiple pathways. Residual confounding by total ischemic time (symptom-to-balloon), infarct territory (anterior vs. non-anterior), and pre-existing coronary disease burden cannot be excluded. Adjustment strategies across included studies were heterogeneous, with many relying on univariable comparisons. The observational nature of all included studies precludes definitive causal inference, and the possibility that collaterals serve primarily as a marker of favorable ischemic preconditioning rather than providing direct myocardial protection should be explicitly acknowledged.

### Clinical implications

Our findings have important implications. The association between collaterals and mortality is prognostic rather than therapeutic while collateral status identifies patients at different risk levels, the LOW certainty of evidence precludes treatment recommendations based on collateral assessment alone. Collateral assessment may be most clinically useful in patients already at elevated risk based on other factors [46–48].

The association between collateral status and outcomes must also be considered in the context of evolving antithrombotic strategies. Across included studies, dual antiplatelet therapy with aspirin and a P2Y12 inhibitor was standard, though specific agents evolved from clopidogrel in earlier studies to predominantly ticagrelor or prasugrel in contemporary cohorts. Notably, patients with robust collaterals who more frequently have multivessel disease may be candidates for complex PCI procedures where periprocedural antithrombotic management, including cangrelor for rapid platelet inhibition, has been shown to reduce ischemic complications [49]. A recent comprehensive review highlights that optimal antithrombotic therapy in complex PCI remains an area of active investigation, with considerations including periprocedural anticoagulation strategies, choice of P2Y12 inhibitor, and duration of dual antiplatelet therapy [50]. Whether antithrombotic intensification strategies differentially benefit patients stratified by collateral status represents an unexplored avenue for future research.

Whether collateral function can be therapeutically enhanced remains speculative. The EXCITE trial demonstrated that exercise training increased collateral flow index by 40% [51], and external counter-pulsation has shown similar benefits [52]. Although the absolute risk reduction of 2.7% and NNT of 37 reflect the prognostic discrimination of collateral status, these metrics should not be interpreted as evidence that treating poor collaterals would prevent one death per 37 patients. Collateral circulation is a biological trait rather than a direct intervention, yet collateral status remains a meaningful prognostic marker even if its relative importance has diminished [53, 54].

### Effect measure interpretation (OR vs. RR)

Although odds ratios were used as the primary effect measure, it is recognized that ORs may slightly overestimate relative risk reductions when event rates are not extremely rare. In the present analysis, the control-group event rate was 6.3%, and the corresponding pooled RR (0.54) was very similar to the pooled OR (0.52), indicating that any potential overestimation is minimal. Accordingly, the observed ‘48% lower odds’ of mortality with robust collaterals corresponds approximately to a 46% relative risk reduction.

### Limitations

Important limitations must be acknowledged. The Rentrop classification has inherent limitations, including interobserver variability approaching 10% and only modest correlation with quantitative measures of collateral function (*r* = 0.32) [55]. Additionally, none of the included studies reported the use of standardized core-laboratory adjudication for collateral grading. Instead, assessments were performed by local investigators with variable expertise and without centralized quality control. This absence of standardized adjudication may introduce nondifferential misclassification of collateral status, which would be expected to bias effect estimates toward the null. Consequently, the true association between collateral status and mortality may be underestimated, although confirmation would require studies using standardized quantitative collateral assessment. The apparent attenuation of the collateral-mortality association in contemporary studies is based on only three studies, each with wide confidence intervals and low event rates, resulting in limited precision and statistical power to detect true effect modification. All included studies were observational, raising concerns about confounding [56]. Although statistical tests did not indicate significant publication bias, funnel plot asymmetry can arise from multiple sources [57]. The 95% prediction interval (0.23–1.18) crossing unity has important implications for external validity. This suggests that in some clinical settings-particularly those with low baseline mortality and highly optimized STEMI care-the true effect of collaterals may include no mortality benefit. Thus, the observed association should be interpreted as context-dependent, highlighting that the relative benefit may vary across different healthcare environments. Finally, Residual confounding by ischemic time, infarct territory, and pre-existing coronary disease cannot be excluded, limiting causal inference.

### Future directions

Quantitative assessment using pressure-derived collateral flow index offers superior precision compared to angiographic grading [58]. Non-invasive CT angiography assessment (sensitivity 92%, specificity 96%) could enable collateral evaluation without invasive angiography [59]. Whether collateral-stratified management strategies could improve outcomes deserves prospective evaluation.

## Conclusions

Robust coronary collateral circulation is associated with significantly lower mortality in STEMI patients undergoing primary PCI. However, this association appears attenuated in contemporary studies, likely reflects statistical dilution due to lower baseline mortality rather than diminished biological relevance of collaterals. Given the LOW certainty of evidence from observational data, collateral assessment should be considered prognostic rather than for a basis for interventions. Future research should evaluate whether therapeutic collateral promotion can improve outcomes in appropriately selected patients.

## Supplementary Information

Supplementary Material 1.

## Data Availability

All data extracted for this systematic review are available in the manuscript and supplementary materials. The study protocol is registered and publicly available at PROSPERO (PROSPERO; Registration ID: CRD420261278634). Statistical code is available from the corresponding author upon reasonable request. No individual patient data were accessed for this analysis.

## Abbreviations

CVD: Cardiovascular disease
STEMI: ST-elevation myocardial infarction
PCI: Percutaneous coronary intervention
MI: Myocardial infarction
MACE: Major adverse cardiovascular events
P2Y12: P2Y12 platelet receptor
MIRON-CL: Myocardial hemorrhage and microvascular obstruction in acute myocardial infarction study
NOS: Newcastle–Ottawa Scale
GRADE: Grading of Recommendations Assessment, Development and Evaluation
