# Supplementary material for: Coronary collateral circulation and mortality in ST-elevation myocardial infarction undergoing primary PCI: an updated systematic review and meta-analysis of 18,443 patients
Source: BMC Cardiovasc Disord. 2026 Mar 20;26:369. doi: 10.1186/s12872-026-05740-w (PMC13126890; doi:10.1186/s12872-026-05740-w)
Supplement: Supplementary file 1 — Supplementary Material 1. [file 12872_2026_5740_MOESM1_ESM.docx]

**SUPPLEMENTARY MATERIALS**

**Table of Contents:**A\ List of Supplementary Tables: (Page 1 – 12) (7 Supplementary Tables)
B\ Supplementary Figures and Figures’ Legends (Page 13 – 18) (6 Supplementary Figures)
C\ List of Supplementary Notes (Page 19) (1 Supplementary Note)

**A\ List of Supplementary Tables**

**Supplementary Table S1.** Complete Search Strategy and Selection Process

| Database / Stage | Search Strategy | Date / Limits | Results (n) |
| --- | --- | --- | --- |
| PubMed/MEDLINE | ((coronary collateral*[tiab]) OR (collateral circulation[tiab]) OR (collateral blood flow[tiab]) OR (collateral vessel*[tiab]) OR (Rentrop[tiab]) OR (collateral connection score[tiab]) OR ("Collateral Circulation"[Mesh])) AND ((STEMI[tiab]) OR (ST elevation myocardial infarction[tiab]) OR (ST-elevation myocardial infarction[tiab]) OR (ST segment elevation myocardial infarction[tiab]) OR ("ST Elevation Myocardial Infarction"[Mesh])) AND ("2020/03/15"[dp] : "2026/01/04"[dp]) | Mar 15, 2020 – Jan 4, 2026 | **59** |
| Embase (Embase.com) | ('coronary collateral*':ti,ab OR 'collateral circulation':ti,ab OR 'collateral blood flow':ti,ab OR 'collateral vessel*':ti,ab OR 'rentrop':ti,ab OR 'collateral connection score':ti,ab OR 'coronary artery collateral circulation'/exp) AND ('stemi':ti,ab OR 'st elevation myocardial infarction':ti,ab OR 'st-elevation myocardial infarction':ti,ab OR 'st segment elevation myocardial infarction':ti,ab OR 'st segment elevation myocardial infarction'/exp) AND [15-03-2020]/sd AND [04-01-2026]/sd NOT [embase]/lim NOT [medline]/lim | Mar 15, 2020 – Jan 4, 2026; exclude Embase + Medline duplicates | **14** |
| Scopus | TITLE-ABS-KEY (("coronary collateral*" OR "collateral circulation" OR "collateral blood flow" OR "collateral vessel*" OR "Rentrop" OR "collateral connection score")) AND TITLE-ABS-KEY (("STEMI" OR "ST elevation myocardial infarction" OR "ST-elevation myocardial infarction" OR "ST segment elevation myocardial infarction")) AND PUBYEAR > 2019 | PUBYEAR 2020–2026 | **131** |
| Total from databases | | | **204** |
| Records from previous meta-analysis (Allahwala et al., 2021)  Studies carried forward from previous review | | | **19** |
| Total records identified (update + previous) | | | **223** |
| Duplicates removed (within update search) | | | **54** |
| Records after duplicates removed (update search) | | | **150** |
| Excluded at title/abstract (update search) | | | **138** |
| Full-text assessed (update search) | | | **12** |
| Excluded with reasons (update search) | | | **5** |
| – Duplicate publication / overlapping cohort | | | **2** |
| – No clinical outcomes | | | **2** |
| – Wrong exposure | | | **1** |
| New studies included (update search) | | | **7** |
| Total studies included in updated meta-analysis  Previous review (19) + new studies (7) | | | **26** |

Comprehensive search strategy applied across three databases (PubMed/MEDLINE, Embase, Scopus) from March 15, 2020 to January 4, 2026. Search terms combined concepts of coronary collateral circulation, STEMI, and primary PCI using Boolean operators. Total unique records identified: 204 (after removing duplicates), combined with 19 studies from previous review for total of 223 records.

**Supplementary Table S2.** Studies Excluded at Full-Text Review

| Study | Year | Reason for Exclusion |
| --- | --- | --- |
| Ying S et al. | 2014 | Duplicate publication (same as Shen 2014) |
| Alsanjari O et al. | 2020 | Duplicate cohort (same as Alsanjari 2019) |
| Zhang Y et al. | 2024 | No clinical outcomes (nomogram study) |
| Chen X et al. | 2021 | Wrong exposure (collaterals to non-IRA CTO) |
| Li W et al. | 2022 | No clinical outcomes (biomarker predictor study) |

Studies were excluded during full-text screening if they met any of the pre-specified exclusion criteria; Duplicate publications or cohorts were excluded to prevent double-counting of participants; Studies without relevant clinical outcomes (mortality, major adverse cardiovascular events) were excluded; CTO = chronic total occlusion; IRA = infarct-related artery; Year indicates publication year; Exclusion decisions were made independently by two reviewers (A.H. & Y.S.) with disagreements resolved through consensus or third-party (S.B.) adjudication.

**Supplementary Table S3.** Full Eligibility Criteria

| - | Criteria |
| --- | --- |
| Inclusion Criteria | 1. Observational studies (prospective or retrospective cohort studies), registry analyses, or randomized controlled trial sub-analyses 2. Studies enrolling patients presenting with ST-elevation myocardial infarction (STEMI) undergoing primary percutaneous coronary intervention (primary PCI) 3. Assessment of coronary collateral circulation to the infarct-related artery prior to reperfusion 4. Evaluation of collateral circulation using angiographic grading, including the Rentrop classification or other clearly defined collateral grading systems 5. Comparison of clinical outcomes between patients with robust collateral circulation (e.g., Rentrop grade 2–3) and poor collateral circulation (e.g., Rentrop grade 0–1) or equivalent classification groups 6. Reporting of at least one relevant clinical outcome, including all-cause mortality, in-hospital mortality, 30-day mortality, long-term mortality, or major adverse cardiovascular events (MACE) 7. Studies involving adult participants aged 18 years or older 8. Full-text articles published in peer-reviewed journals |
| Exclusion Criteria | 1. Case reports or case series 2. Review articles, systematic reviews, meta-analyses, editorials, commentaries, letters, or expert opinion articles 3. Conference abstracts without subsequent full-text publication or insufficient data for extraction 4. Animal or preclinical studies 5. Studies focusing exclusively on coronary collateral circulation in chronic total occlusions unrelated to the infarct-related artery 6. Studies without clinical outcome data or insufficient outcome reporting 7. Pediatric populations (participants younger than 18 years) 8. Duplicate publications or overlapping study populations (most complete or most recent dataset retained) 9. Studies where collateral circulation was assessed after reperfusion therapy 10. Studies lacking clear definitions or stratification of collateral circulation status |

**Supplementary Table S4.** Per-study mortality data used in the primary meta-analysis (standard definition set)

| Study | Robust collaterals (deaths/N) | Poor collaterals (deaths/N) | OR (95% CI)* | Outcome timepoint used |
| --- | --- | --- | --- | --- |
| Elsman 2004 | 3/106 | 43/953 | 0.62 (0.19–2.02) | 1-year mortality |
| Sorajja 2007 | 3/119 | 8/199 | 0.62 (0.16–2.37) | 6-month mortality |
| Desch 2010 | 3/69 | 22/166 | 0.30 (0.09–1.03) | Long-term mortality |
| Rechcinski 2013 | 7/78 | 21/242 | 1.04 (0.42–2.54) | Long-term mortality |
| Kajiya 2014 | 0/33 | 4/31 | 0.09 (0.00–1.77) | 4-year cardiac death |
| Shen 2014 | 2/54 | 20/193 | 0.53 (0.12–2.34) | 6-month mortality |
| Hara 2016 | 44/770 | 183/2570 | 0.53 (0.39–0.71) | In-hospital mortality |
| Kim 2016 | 1/60 | 0/329 | 10.85 (0.44–270.18) | 1-year mortality |
| Sen 2017 | 5/278 | 58/1097 | 0.33 (0.13–0.83) | 30-day mortality |
| Hernandez-Perez 2017 | 14/212 | 91/735 | 0.50 (0.28–0.90) | Long-term mortality |
| Chu 2019 | 2/108 | 17/238 | 0.25 (0.06–1.08) | In-hospital mortality |
| Alsanjari 2019 | 10/357 | 122/1587 | 0.35 (0.18–0.67) | In-hospital mortality |
| Allahwala 2020 | 7/345 | 97/1280 | 0.25 (0.12–0.55) | In-hospital mortality |
| Freund 2020 | 2/33 | 16/62 | 0.19 (0.04–0.86) | Long-term mortality |
| Liu 2020 | 4/323 | 26/1339 | 0.63 (0.22–1.83) | In-hospital mortality |
| Iqbal 2024 | 3/102 | 9/245 | 0.79 (0.21–3.00) | 30-day mortality |
| Yilmaz 2025 | 8/272 | 24/908 | 1.12 (0.50–2.51) | In-hospital mortality |

Individual study mortality data for 17 studies using standard Rentrop classification (grade 2-3 vs 0-1) in primary analysis. Total events: 118/3,319 (3.6%) in robust collateral group vs 761/12,174 (6.3%) in poor collateral group. Follow-up periods ranged from in-hospital to long-term (≥1 year). OR calculated using random-effects model.

**Supplementary Table S5.** Exploratory Subgroup Findings

| Subgroup by study finding | | | | | p=0.05 |
| --- | --- | --- | --- | --- | --- |
| Protective | 10 | 0.43 | 0.28-0.65 | 53% | <0.0001 |
| NULL | 7 | 0.77 | 0.50-1.16 | 0% | 0.21 |

An exploratory subgroup analysis by authors' reported conclusions showed that studies concluding a significant association (n=10) had OR 0.43 (95% CI 0.28-0.65), while studies reporting null findings (n=7) had OR 0.77 (95% CI 0.50-1.16; p=0.05 for subgroup difference). However, this analysis is limited by classification based on authors’ study conclusions rather than predefined objective criteria.

**Supplementary Table S6.** Meta-Regression Results

| Covariate | Coefficient | 95% CI | p-value | R^2^ % |
| --- | --- | --- | --- | --- |
| Publication year | -0.001 | -0.05 to 0.05 | 0.993 | 0 |
| Sample size (per 100) | 0.002 | -0.004 to 0.008 | 0.543 | 0 |
| Control event rate (%) | -0.04 | -0.08 to -0.002 | 0.038 | 19.2 |

**Supplementary Table S7.** Risk of Bias Assessment (Newcastle-Ottawa Scale)

| Study | Selection (0-4) | Comparability (0-2) | Outcome (0-3) | Total (0-9) | Rating |
| --- | --- | --- | --- | --- | --- |
| Perez-Castellano 1998 | 4 | 2 | 3 | 9 | Good |
| Antoniucci 2002 | 4 | 2 | 3 | 9 | Good |
| Elsman 2004 | 4 | 1 | 3 | 8 | Good |
| Sorajja 2007 | 3 | 1 | 3 | 7 | Good |
| Desch 2010 | 4 | 2 | 3 | 9 | Good |
| Wang 2011 | 4 | 1 | 2 | 7 | Good |
| Valim 2011 | 4 | 0 | 3 | 7 | Good |
| Rechcinski 2013 | 4 | 1 | 3 | 8 | Good |
| Kajiya 2014 | 3 | 0 | 3 | 6 | Fair |
| Shen 2014 | 4 | 2 | 2 | 8 | Good |
| Yaylak 2015 | 3 | 1 | 3 | 7 | Good |
| Hara 2016 | 4 | 2 | 3 | 9 | Good |
| Kim 2016 | 4 | 2 | 3 | 9 | Good |
| Sen 2017 | 4 | 1 | 3 | 8 | Good |
| Hernandez-Perez 2017 | 4 | 1 | 3 | 8 | Good |
| Chu 2019 | 4 | 2 | 2 | 8 | Good |
| Alsanjari 2019 | 4 | 2 | 3 | 9 | Good |
| Allahwala 2020 | 4 | 2 | 3 | 9 | Good |
| Freund 2020 | 3 | 2 | 3 | 8 | Good |
| Liu 2020 | 4 | 1 | 2 | 7 | Good |
| Park 2021 | 4 | 2 | 1 | 7 | Good |
| Pec 2023 | 3 | 0 | 3 | 6 | Fair |
| Scholz 2023 | 4 | 2 | 3 | 9 | Good |
| Iqbal 2024 | 4 | 0 | 3 | 7 | Good |
| Yılmaz 2025 | 4 | 1 | 3 | 8 | Good |
| Vora 2025 | 4 | 2 | 3 | 9 | Good |


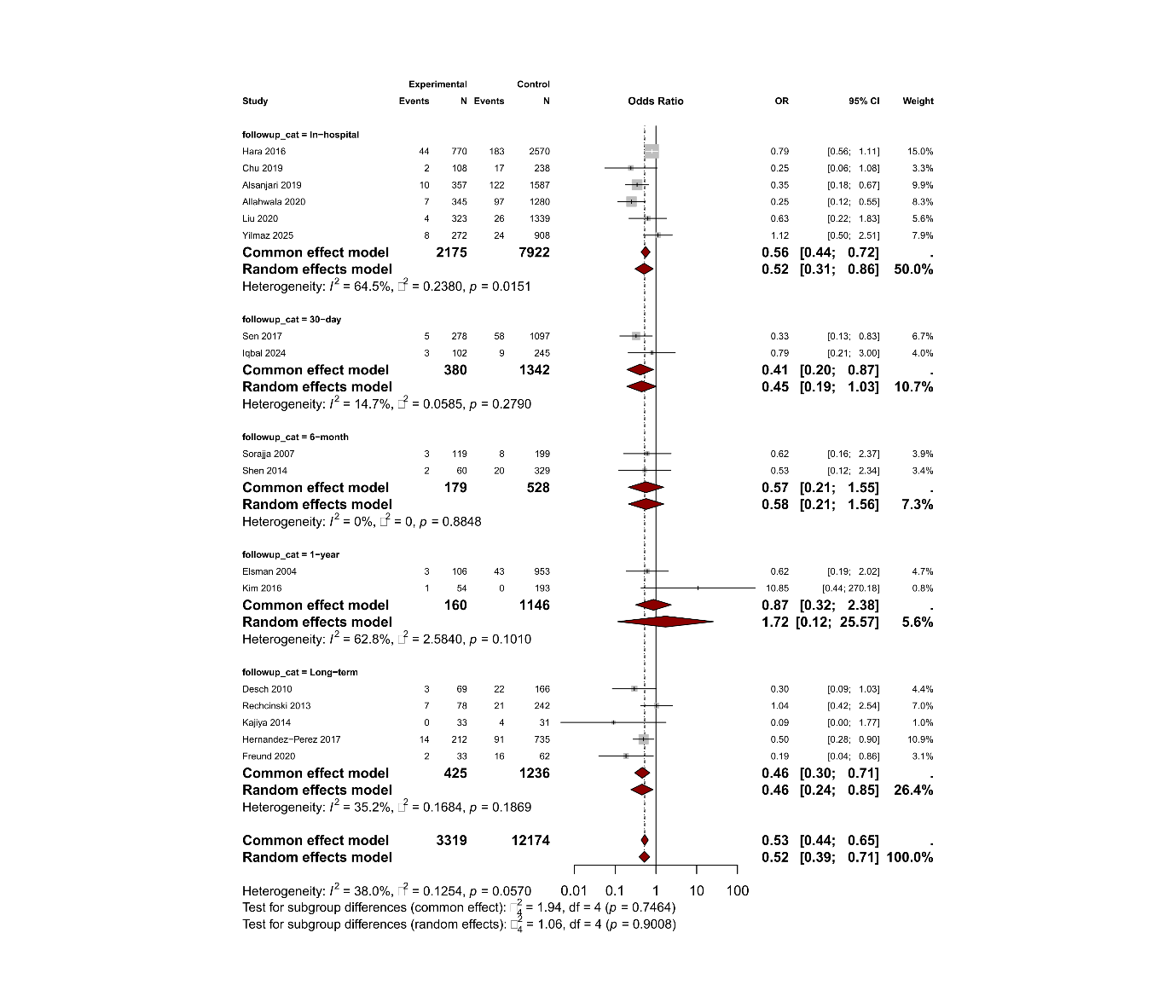
**B\ Supplementary Figures and Figures’ Legends**

**Supplementary Figure S1. Subgroup analysis by follow-up duration:** Forest plots stratified by follow-up timepoints: in-hospital (OR 0.52, n=6 studies), 30-day (OR 0.45, n=2), 6-month (OR 0.58, n=2), 1-year (OR 1.72, n=2), and long-term >1 year (OR 0.46, n=5). Test for subgroup differences showed no significant interaction (p=0.90), indicating consistent protective effects across all follow-up durations.


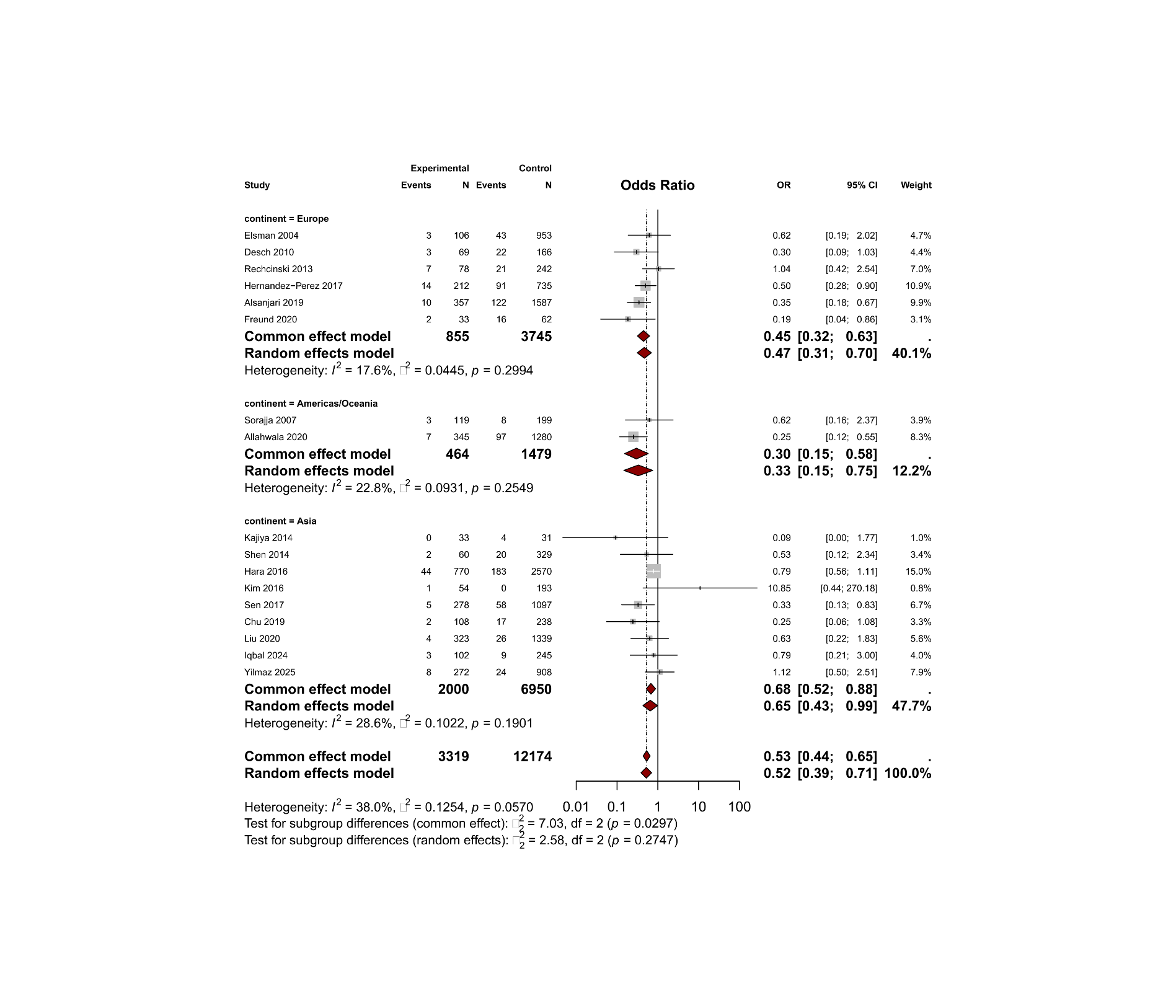
**Supplementary Figure S2. Subgroup analysis by geographic region:** Subgroup analysis by continent showing consistent protective effects: Europe (OR 0.47, 95% CI 0.31-0.70, n=6 studies), Americas/Oceania (OR 0.33, 95% CI 0.15-0.75, n=2), and Asia (OR 0.65, 95% CI 0.43-0.99, n=9). Test for subgroup differences non-significant (p=0.27), demonstrating geographic consistency of collateral benefit.


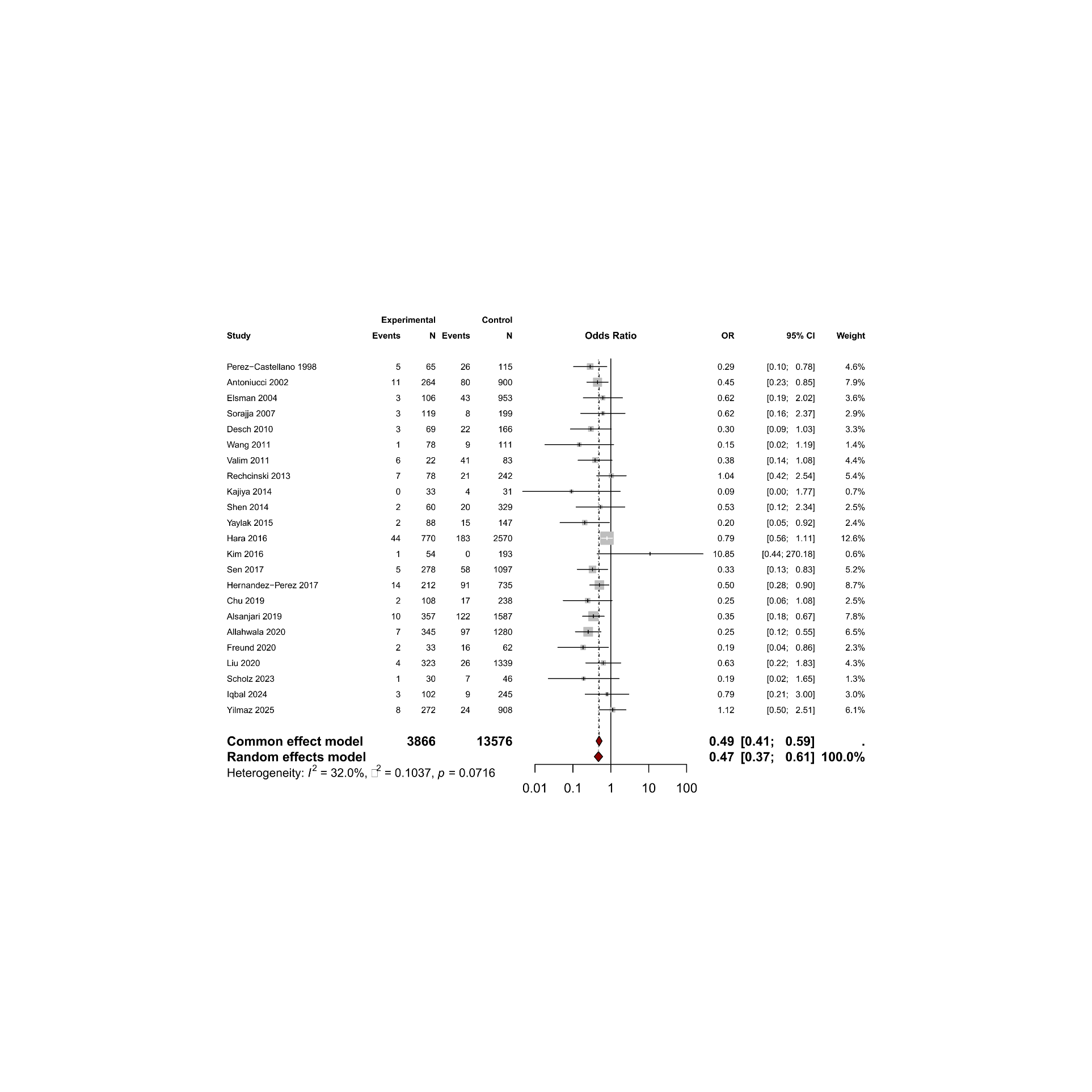
**Supplementary Figure S3. Forest plot of sensitivity analysis including all studies (n=23):** Sensitivity analysis including all 23 studies regardless of collateral definition (standard and non-standard Rentrop classifications). Pooled random-effects OR 0.47 (95% CI 0.37-0.61), with moderate heterogeneity (I²=32.0%, p=0.037). Results remained consistent with primary analysis, confirming robustness of findings across different collateral classification schemes.


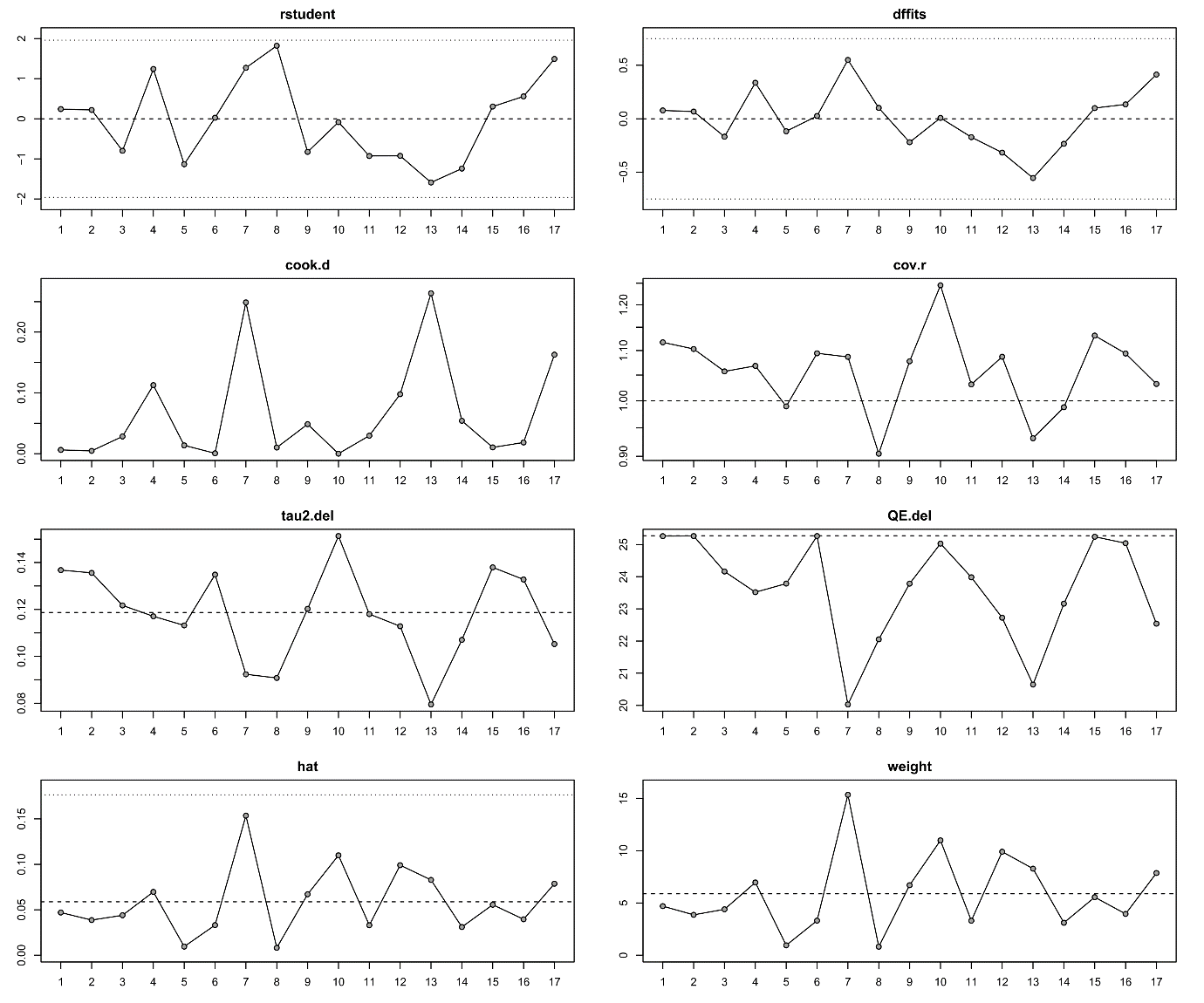
 **Supplementary Figure S4. Influence diagnostics (leave-one-out influence analysis):** Leave-one-out sensitivity analysis showing pooled OR estimates after sequentially removing each study. Results ranged from 0.49 to 0.73, with all estimates remaining statistically significant, demonstrating robustness of findings. No single study exerted disproportionate influence on overall effect estimate, confirming stability of meta-analytic results.


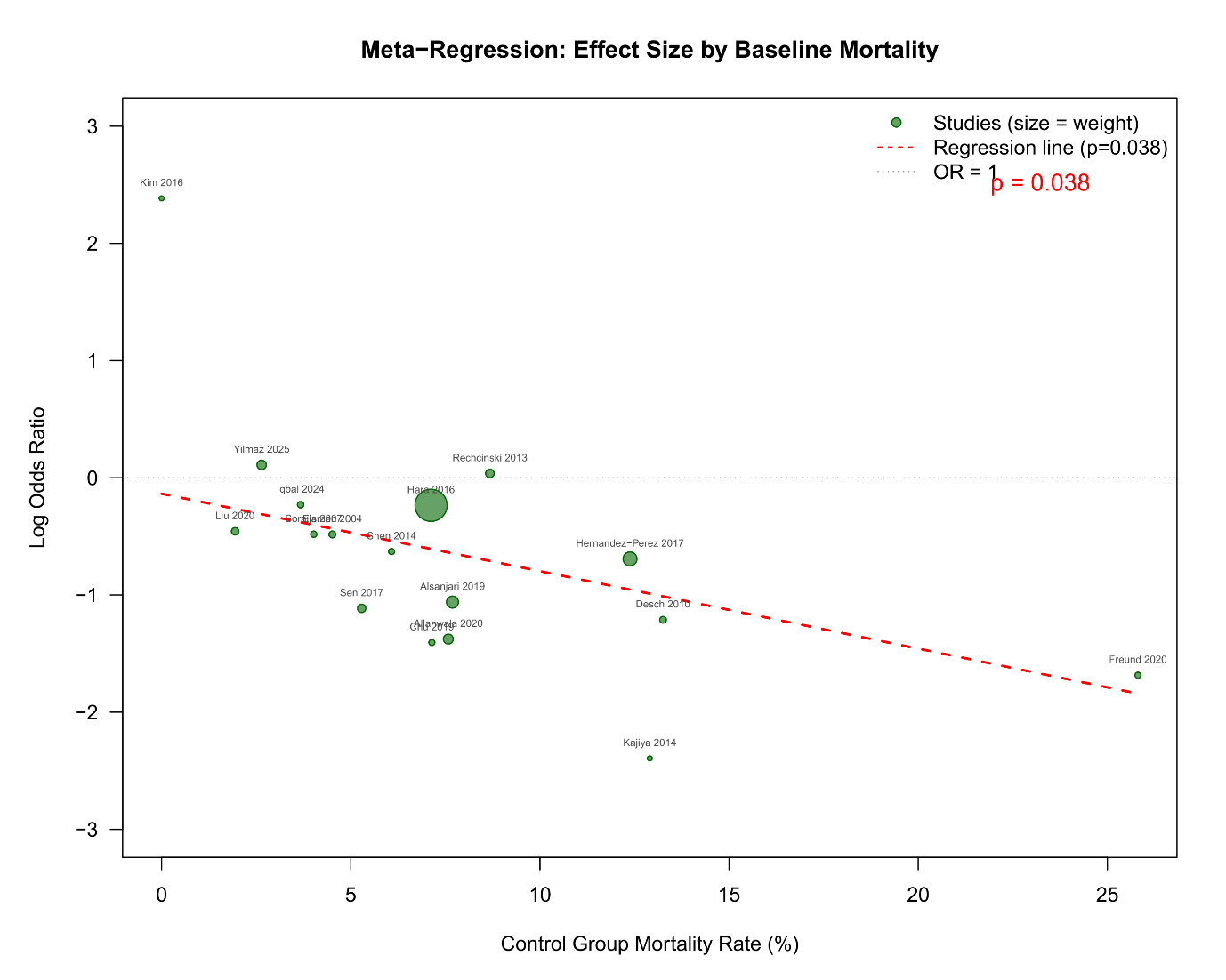
 **Supplementary Figure S5. Bubble plot of meta-regression (effect size vs. control event rate):** Meta-regression showing significant negative association between control group mortality rate and log odds ratio (coefficient -0.04, p=0.038). Studies with higher baseline mortality (right side) demonstrated larger protective effects from robust collaterals, suggesting greater absolute benefit in higher-risk populations. Bubble size represents study weight.


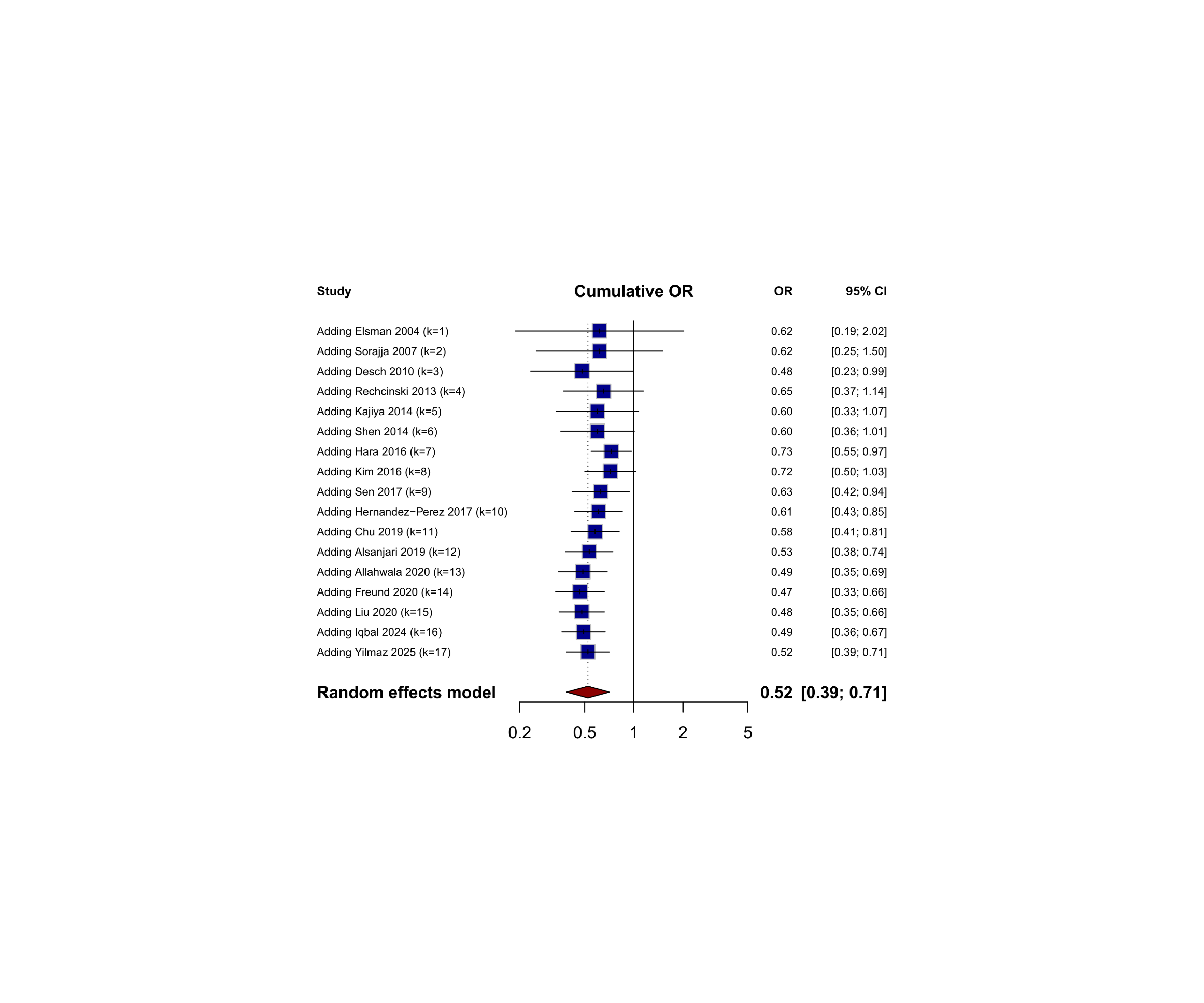
 **Supplementary Figure S6. Cumulative meta-analysis by publication year:** Cumulative Forest plot showing sequential addition of studies from 2004 to 2025. Effect estimate remained stable over time around OR 0.50-0.60, with progressive narrowing of confidence intervals as evidence accumulated. Final pooled estimate OR 0.52 (95% CI 0.39-0.71) maintained significance throughout cumulative addition.

**C\ List of Supplementary Notes**

**Supplementary Note 1. Trial Sequential Analysis**

Trial sequential analysis (TSA) with α=0.05 (two-sided), β=0.20 (80% power), control event rate of 6.3%, and relative risk reduction of 46% calculated a required information size of 1,724 patients. With 15,493 patients included, the analysis exceeds 899% of required information size.

**Important caveats:** TSA was developed for sequential monitoring of randomized controlled trials and its application to observational meta-analysis has important limitations: (1) the assumed effect size (46% RRR) is derived from the observed pooled estimate, introducing circularity; (2) observational studies are subject to confounding that TSA cannot address; (3) the precision implied by TSA may be spurious given heterogeneity in study populations and methods. This analysis should be interpreted as suggesting that random error is unlikely to explain the observed association, rather than as definitive evidence of a true causal effect.
